# Tram-track-like calcification in adult optic pathway glioma

**DOI:** 10.1259/bjrcr.20150378

**Published:** 2016-07-28

**Authors:** Amalraj Sindhuja, Venkatraman Indiran, Rengarajan Santhanam, Prabakaran Maduraimuthu

**Affiliations:** ^1^Department of Radiodiagnosis, Sree Balaji Medical College and Hospital, Chennai, India; ^2^Department of Neurosurgery, Sree Balaji Medical College and Hospital, Chennai, India

## Abstract

Gliomas of the optic pathways that occur in adults are rare tumours. The tram-track-like calcification of the optic nerve, which classically occurs with meningioma, has not been reported so far in optic pathway gliomas. Here, we present a case of high-grade glioma of the optic pathway with tram-track-like calcification of the optic nerve in a 42-year-old male. This case enhances our understanding of the MRI features of adult malignant optic pathway glioma.

## Case report

A 42-year-old male was brought to our emergency room with an episode of loss of consciousness for half hour. The patient had been complaining of headache, blurring of vision in the left eye and vomiting for the past 1 month. He was drowsy, confused and irritable. His general physical examination was normal. He had relative afferent pupillary defect in the left eye. Optic fundus examination showed bilateral papilloedema. Visual assessment performed subsequently showed no perception of light in the left eye and a visual acuity of 6/24 in the right eye.

**Figure 1.**
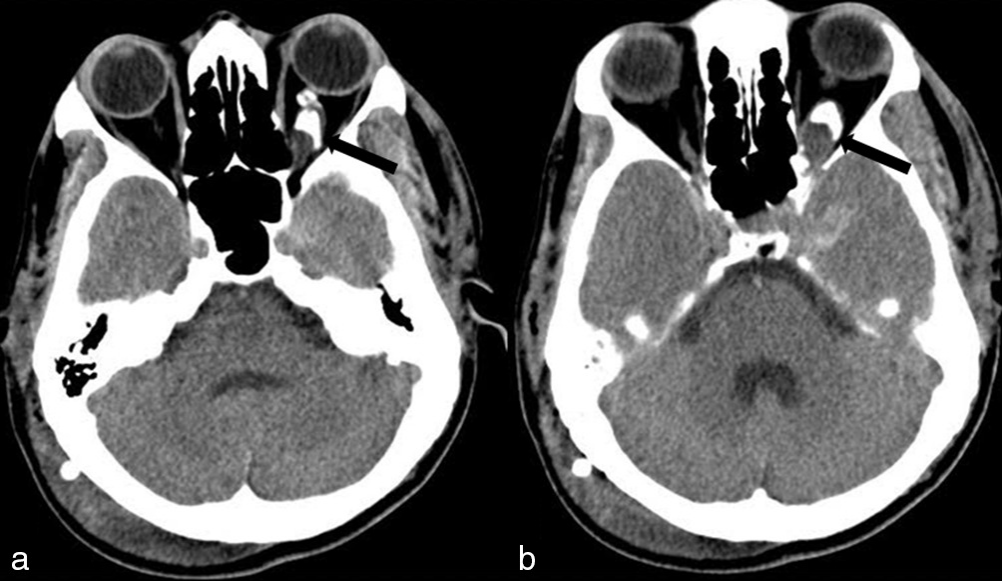
Axial non-contrast CT images. (a, b) The optic nerve sheath complex appeared thickened and tortuous and showed peripheral tram-track-like calcification with a widened optic canal (black arrows).

CT scan showed a large heterogeneous ill-defined lesion in the suprasellar region, which extended into the left basifrontal region and through the left optic canal into the left orbit. The optic nerve sheath complex appeared thickened and tortuous, and showed peripheral tram-track-like calcification with a widened optic canal. Minimal hydrocephalus was seen (Figure 1).

**Figure 2.**
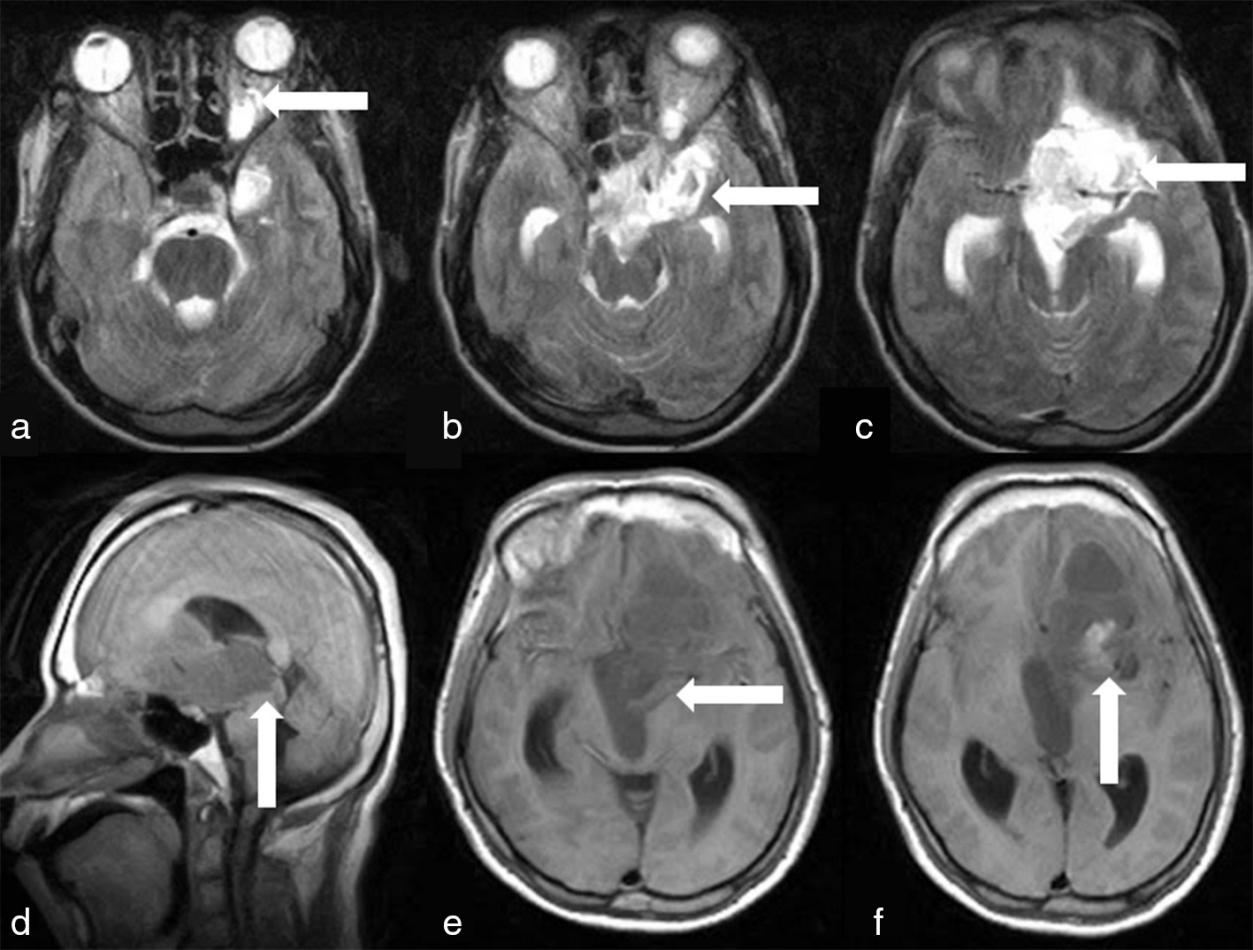
Axial *T*_2_ weighted images (a–c) show a large heterogeneous ill-defined lesion in the suprasellar region, extending superiorly into the left basifrontal region and anteriorly through the left optic canal into the left orbit. The left optic nerve appeared thickened (white arrows). *T*_1_ weighted sagittal image (d) and *T*_1_ weighted axial images (e, f) show the lesion exerting significant mass effect on the anterior aspect of the left lateral ventricle and the floor of the third ventricle (white arrows), causing obstructive hydrocephalus and cerebral oedema.

**Figure 3.**
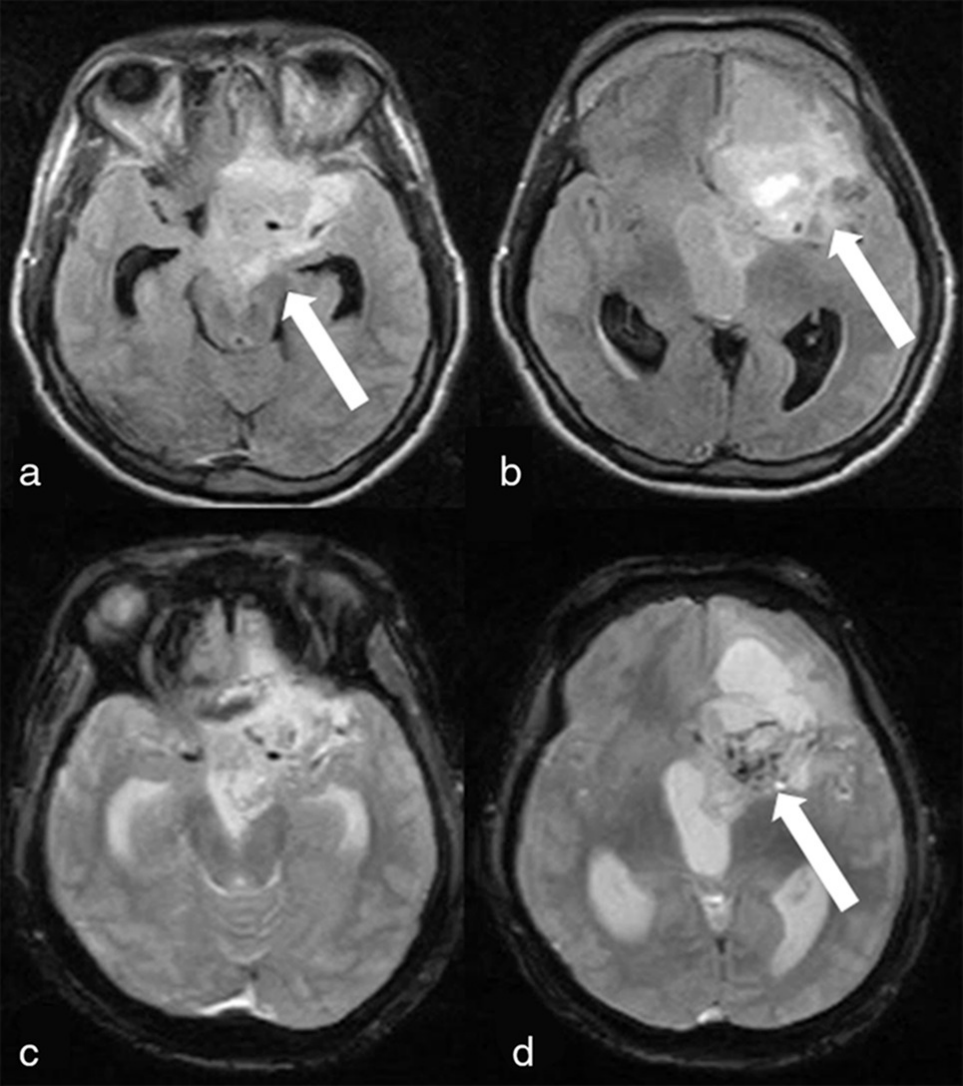
*T*_2 _weighted fluid-attenuated inversion-recovery images (a, b) and gradient recalled echo images (c, d) show a large heterogeneous ill-defined lesion in the suprasellar region, extending into the interpeduncular cistern (white arrow in a) with hyperintense haemorrhagic foci (white arrow in b) and gradient recalled echo hypointense haemorrhagic foci in the left frontal region (white arrow in d).

MRI of the brain showed a large heterogeneous ill-defined lesion seen in the suprasellar region, which extended superiorly into the left basifrontal region and anteriorly through the left optic canal into the left orbit. The left optic nerve appeared thickened and showed tram-track calcification. The optic tract and the chiasma were not seen separately from the lesion. The lesion was seen exerting significant mass effect on the anterior aspect of the left lateral ventricle and the floor of the third ventricle, causing obstructive hydrocephalus and cerebral oedema. Posteriorly, the lesion was seen extending into the interpeduncular cistern (Figures 2 and 3). The patient suffered a seizure during the MRI; hence, contrast-enhanced MRI could not be performed. Diagnoses of optic pathway glioma and meningioma were considered.

The patient was taken up for emergency surgery. Left frontal craniotomy with decompression of the tumour was performed and sample was sent for histopathology.

**Figure 4.**
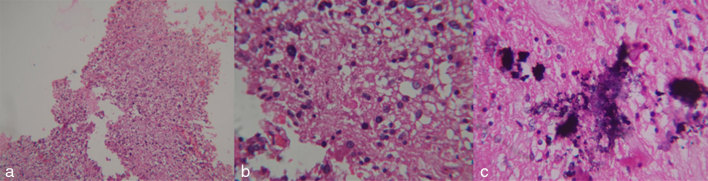
Haematoxylin and eosin-stained slides (a, low power; b, high power; c, calcification within the tumour) showing fragments of glial tissue enclosing a neoplasm composed of round to oval cells with enlarged nucleus and perinuclear halo traversed by thin-walled blood vessels. Some fragments show collection of gemistocytes and astrocytes with dark staining pleomorphic nuclei. Stroma shows hyalinized blood vessels, microcystic degeneration and foci of calcification.

The tumour was very vascular and had variable consistency with areas of gritty calcification. Areas of necrosis filled with dark brown-coloured fluid were seen. A ventriculoperitoneal shunt tube was placed for relieving the hydrocephalus. He was then treated with intensity-modulated radiotherapy and chemotherapy with temozolomide. The lesion turned out to be an anaplastic oligoastrocytoma on histopathological examination (Figure 4).

## Discussion

Gliomas account for 40–50% of all intracranial tumours. Glioblastoma is the most common type of glioma.^1^ World Health Organization (WHO) Grade I and II astrocytomas are usually well defined and homogenous and show less mass effect and oedema, whereas WHO Grade III anaplastic astrocytomas are less clearly delineated and show moderate mass effect, oedema and heterogeneity, and exhibit variable enhancement. WHO Grade IV glioblastomas are poorly defined and show extensive mass effect, oedema, haemorrhage and heterogeneity.^2^

Gliomas of the optic pathway are classified into two groups—the relatively benign optic nerve glioma (typically occurring in the paediatric age group) and the malignant optic glioma of adulthood.^3^ Optic pathway gliomas are more common in children and occur in a setting of neurofibromatosis Type 1 (NF-1). In this setting, it is often low grade and indolent.^4^ Bilateral optic nerve gliomas are almost pathognomonic for NF-1. In adults, it is a relatively rare optic pathway tumour and was thought to be usually aggressive and associated with high mortality rate. In such cases, no association with NF-1 is found.^5^ However, in a study involving 22 adults with optic gliomas, Shofty et al^6^ found that not all optic pathway gliomas were aggressive. They divided patients with adult optic pathway gliomas into those with tumours diagnosed during childhood (12/22) and those in whom tumours were diagnosed during adulthood (10/22). Six of the 10 patients diagnosed at adulthood demonstrated visual deterioration with a concomitant progression in five of them. Only two of them were diagnosed to have high grade gliomas.^6^

In a review of the literature in 2004 by Wabbles et al,^7^ 45 cases of adult malignant optic gliomas were described. 51% of the patients were males and 49% were females, with the mean age at diagnosis being 54 years. They demonstrated that malignant optic glioma could also arise at the hypothalamus (50% of patients), the temporal lobe (22.5% of patients) and the basal ganglia (15% of patients) apart from the typical sites at the optic chiasma and optic nerve(s).^7^ No definite association between neurofibromatosis and adult optic glioblastoma has been described. Histopathologically, malignant optic gliomas of adulthood are classified as either anaplastic astrocytoma or glioblastoma multiforme.^3^

Calcification is a common characteristic in optic nerve sheath meningioma. CT scan is more sensitive than MRI in identifying calcification. Optic nerve sheath meningiomas show homogeneous enhancement around a hypointense optic nerve, giving the tram-track sign.^8^ Similarly, on non-contrast CT images, linear calcification of the nerve in optic nerve sheath meningiomas is also suggestive of tram-track appearance.^9^

In malignant optic glioma of adulthood, progressive loss of visual acuity and monocular blindness occur in rapid succession, as seen in our case.

Optic nerve gliomas of adulthood may be treated with surgery, radiation and chemotherapy. In spite of various treatment options that are available, the prognosis remains poor, with a mean survival time of less than a year.^10^ Our patient has been on regular follow-up for the past 6 months and has been seizure-free during this period.

## Learning points

1. Optic pathway gliomas occurring in adulthood have higher chances of being malignant.
2. Optic pathway gliomas occurring in adulthood are not associated with neurofibromatosis.
3. The tram-track calcification classically seen in optic pathway meningiomas can very rarely be seen in optic pathway gliomas as well.

## Consent

Patient identity information is not revealed here. Informed consent was obtained.
